# Use of Flavin-Containing Monooxygenases for Conversion of Trimethylamine in Salmon Protein Hydrolysates

**DOI:** 10.1128/AEM.02105-20

**Published:** 2020-11-24

**Authors:** Marianne Goris, Pål Puntervoll, David Rojo, Julie Claussen, Øivind Larsen, Antonio Garcia-Moyano, David Almendral, Coral Barbas, Manuel Ferrer, Gro Elin Kjæreng Bjerga

**Affiliations:** a NORCE Norwegian Research Centre, Bergen, Norway; b Centre for Metabolomics and Bioanalysis (CEMBIO), Department of Chemistry and Biochemistry, Facultad de Farmacia, Universidad San Pablo-CEU, CEU Universities, Urbanización Montepríncipe, Madrid, Spain; c Institute of Catalysis, Consejo Superior de Investigaciones Científicas (CSIC), Madrid, Spain; Shanghai Jiao Tong University

**Keywords:** enzyme discovery, fish protein hydrolysate, flavin-containing monooxygenases, malodor, trimethylamine, trimethylamine *N*-oxide, trimethylamine monooxygenases, oxidoreductases

## Abstract

Enzymatic processing of fish by-products for recovery of peptides (hydrolysates) is a promising technology to reach food grade ingredients of high nutritional quality. Despite this, their bitter taste and “fish” odor block implementation in food products and limit their economic potential. Trimethylamine (TMA) is a known contributor to malodor in fish. Current strategies to mask or remove the odor either are not effective or give rise to undesirable side effects. As an alternative approach to remediate TMA, we propose a novel enzymatic strategy to convert TMA into the odorless trimethylamine *N*-oxide (TMAO) using TMA monooxygenases (Tmms). We identified a diverse set of bacterial Tmms using a sequence similarity network. Purified, recombinant enzymes were assessed for their biocatalytic capacity by monitoring NADPH consumption and TMAO generation. Selected Tmms were subjected to biochemical characterization and investigated for their ability to oxidize TMA in an industry-relevant substrate. From the 45 bacterial Tmm candidates investigated, eight enzymes from four different taxa were selected for their high activity toward TMA. The three most active enzymes were shown to vary in temperature optimum, with the highest being 45°C. Enzymatic activity dropped at high temperatures, likely due to structural unfolding. The enzymes were all active from pH 6.0 to 8.5, with functional stability being lowest around the optimal pH. All three Tmms, given sufficient NADPH cofactor, were found to generate TMAO in the TMA-rich salmon protein hydrolysate. The Tmms serve as unique starting points for engineering and should be useful for guiding process development for marine biorefineries.

**IMPORTANCE** Enzyme-based conversion of marine biomass to high-quality peptide ingredients leaves a distinct smell of “fish” caused by the presence of trimethylamine, which limits their economic potential. We suggest an enzymatic solution for converting trimethylamine to the odorless trimethylamine *N*-oxide as a novel strategy to improve the smell quality of marine protein hydrolysates. Following a systematic investigation of 45 putative bacterial trimethylamine monooxygenases from several phyla, we expand the repertoire of known active trimethylamine monooxygenases. As a proof-of-concept, we demonstrate that three of these enzymes oxidized trimethylamine in an industry-relevant salmon protein hydrolysate. Our results add new oxidoreductases to the industrial biocatalytic toolbox and provide a new point of departure for enzyme process developments in marine biorefineries.

## Abstract (precis)

Enzyme-based conversion of marine biomass to high-quality peptide ingredients leaves a distinct smell of “fish” caused by the presence of trimethylamine, which limits their economic potential. We suggest an enzymatic solution for converting trimethylamine to the odorless trimethylamine *N*-oxide as a novel strategy to improve the smell quality of marine protein hydrolysates. Following a systematic investigation of 45 putative bacterial trimethylamine monooxygenases from several phyla, we expand the repertoire of known active trimethylamine monooxygenases. As a proof-of-concept, we demonstrate that three of these enzymes oxidized trimethylamine in an industry-relevant salmon protein hydrolysate. Our results add new oxidoreductases to the industrial biocatalytic toolbox and provide a new point of departure for enzyme process developments in marine biorefineries.

## INTRODUCTION

The processing of fish for human consumption gives rise to 50 to 70% by-products, such as heads, frames, viscera, blood, and trimmings (1), leading to an estimated 1 million tons of by-products annually in Norway alone (2). In recent decades, there has been great progress in the development of enzyme-based processes by which fish by-products are converted into marine ingredients, such as peptides and oils. Today, these ingredients are mainly used to manufacture animal and pet feed, but since the by-products contain high-quality proteins, they also have a great potential for human consumption. Depending on processing conditions, however, enzymatically derived fish protein hydrolysates may suffer from a distinct malodor (3), which is described as an unpleasant smell associated with rotting fish. This currently limits their potential in the human consumption market.

Trimethylamine (TMA) is a major contributor of malodor from fish and is recognizable as a pungent fish odor (4). Even very low TMA levels, down to 0.00021 ppm, have been reported as recognizable by humans (5). In fish, TMA accumulates postmortem as a result of bacterial conversion of the oxygenated odorless precursor, trimethylamine *N*-oxide (TMAO) (6). TMAO acts as an osmolyte that stabilizes proteins during environmental stress, such as osmotic pressure, and it is therefore believed to be particularly abundant in species located in the depths of the ocean (7). As TMA is common in marine environments, a possible enzymatic solution to this malodor challenge may be found in marine bacteria that can utilize TMA as their sole carbon and/or nitrogen source, where the first step is to convert TMA to odorless TMAO (8).

The oxidation of TMA to TMAO is catalyzed by monooxygenases, which, as reflected by their name, are enzymes that catalyze the insertion of a single oxygen atom into their substrates. Several classes of monooxygenases have been described (9). Class B flavin-dependent monooxygenases include class I Baeyer Villiger monooxygenases (BVMOs), *N*-hydroxylating monooxygenases (NHMOs), YUCCAs, and flavin-containing monooxygenases (FMOs), and together they are involved in several key biological processes (10, 11). Structurally, class B flavin-dependent monooxygenases contain two Rossmann fold domains that harbor dinucleotide binding motifs, namely, for their tightly bound flavin adenine dinucleotide (FAD) and NADP (NADPH) cofactors, on which the enzymes depend for their catalytic action (10). The TMA-oxidizing monooxygenases are classified as FMOs, and both mammalian and bacterial FMOs appear to utilize a “cocked-gun” mechanism (12, 13), where FAD is reduced by NADPH in the absence of substrate, generating the reactive intermediate C4a-hydroperoxy-FAD and NADP^+^. The C4a-hydroperoxy-FAD intermediate is stabilized and shielded by NADP^+^ (13, 14) and readily inserts one oxygen atom into the substrate upon entering the active site. Whether the NADP^+^ remains bound to the active site during catalysis has been debated (13–15).

FMO enzymes that convert TMA to TMAO have been named trimethylamine monooxygenases (Tmms) (16); they are promiscuous enzymes, found in all kingdoms of life (12, 16–18), that can oxidize a range of additional nonpolar substrates, such as indole and methimazole (13, 16). The most studied Tmm is the human hepatic enzyme FMO3, which is responsible for TMA-to-TMAO conversion in the liver (19). Malfunction of FMO3 is linked to the metabolic disorder trimethylaminuria, or fish odor syndrome. Patients suffering from this disease are unable to rid their body of TMA, leading to a strong bodily odor resembling that of spoiled fish (20).

Tmms have been found to be abundant in marine bacterial metagenomes, suggesting that they play a crucial role in the carbon and nitrogen cycling in the ocean (16). The first bacterial Tmm, mFMO, was discovered in Methylophaga aminisulfidivorans strain SK1, which is marine and belongs to the *Gammaproteobacteria* (13, 14, 18). Other marine bacterial Tmms shown to be active have been found in the *Alphaproteobacteria* and include RnTmm from Roseovarius nubinhibens strain ISM (15) and Tmms from Methylocella silvestris strain BL2, *Roseovarius* sp. strain 217, Ruegeria pomeroyi strain DSS-3, Pelagibacter ubique strain HTCC1002, and Pelagibacter ubique strain HTCC721 (16). Tmms have also been described in other habitats, including NiFMO from Nitrincola lacisaponensis, which was isolated from an alkaline saline lake (21), and cFMO from Corynebacterium glutamicum, isolated from soil (22). The fact that C. glutamicum belongs to the *Actinobacteria* suggests that there may be Tmms of potential industrial interest outside the *Proteobacteria*.

The aim of this study was to identify bacterial Tmms that efficiently convert TMA to TMAO when expressed recombinantly in Escherichia coli and to test their ability to perform this catalytic reaction in a TMA-rich salmon protein hydrolysate. To this end, a sequence similarity network (SSN) analysis was employed to select 45 Tmm candidates from six taxonomic groups. Successfully expressed and soluble candidates were purified and assessed for their ability to convert TMA to TMAO by monitoring both NADPH cofactor consumption and TMAO product formation. As a first step toward industrial application, the three best-performing Tmms were characterized biochemically, by studying their temperature and pH profiles, as well as by assessing their structural and functional stability. Finally, as a proof of concept, we demonstrate that our top three Tmms were all able to convert TMA to TMAO in a salmon protein hydrolysate.

## RESULTS

### Trimethylamine monooxygenase candidate selection.

**FIG 1:**
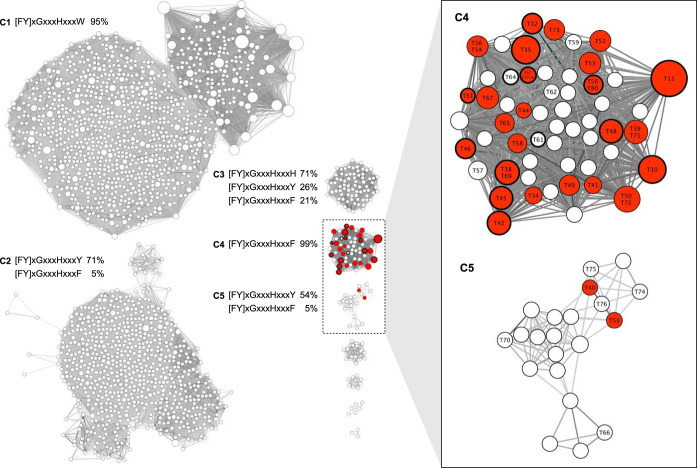
FMO-like family sequence similarity network (SSN). EFI-EST was used to generate a representative node SSN of all UniRef90 FMO-like family (IPR020946) sequences, where each node is a collection of proteins with 40% or higher identity. Nodes containing only nonbacterial sequences and edges with alignment scores lower than 50 were removed, resulting in the depicted SSN consisting of nine clusters. The five largest clusters were named C1 to C5, and the presence and abundance of BVMO (C1) and FMO signature (C2 to -5) motifs within sequences of those clusters are indicated. Nodes with at least one trimethylamine monooxygenase (Tmm) annotated sequence are shown in red and were present in clusters C4 and C5. Candidate Tmm sequences selected for recombinant expression are indicated by their arbitrary identification (ID) number (T10 to T80) within their respective nodes, and nodes containing at least one sequence which was successfully expressed and showed detectable Tmm activity are highlighted by bold circles. Node sizes reflect the numbers of sequences contained within (smallest, 1, and largest, 109), and edges are drawn in shades of gray, reflecting alignment scores (a darker shade means a higher score).

To identify new bacterial Tmms, we performed an SSN analysis using the Enzyme Function Initiative-Enzyme Similarity Tool (EFI-EST) (23). Although the known Tmms belong to the flavin monooxygenase (FMO) family (InterPro accession number IPR000960), we expanded the search to the encompassing FMO-like family (InterPro accession number IPR020946) to ensure that all relevant sequences were included. The SSN analysis was thus performed on all 19,736 FMO-like family UniRef90 sequences, resulting in a representative node network of 6,740 nodes, where each node represents a collection of proteins with 40% or higher identity. Nodes containing only nonbacterial sequences and edges with alignment scores lower than 50 were removed, resulting in a network of nine distinct clusters containing a total of 1,927 nodes (Fig. 1). The largest cluster, C1, contains 1,036 nodes, representing 11,652 sequences, of which 95% contain the BVMO signature motif [FY]XGXXXHXXXW, first published as FXGXXXHXXXW[PD] (24). Although there are also sequences annotated as BVMOs in cluster C2, none of the sequences outside C1 contain the BVMO signature motif, suggesting the SSN analysis successfully separated the BVMO sequences from the FMO sequences, which have the signature motif (FY)XGXXXHXXX(FY), first published as FXGXXXHXXX(FY) (24). The occurrence and abundance of variants of the FMO signature motif in the four largest FMO family sequence clusters, C2 to -5, are given in Fig. 1. The FMO signature motif variant (FY)XGXXXHXXXY dominated the C2 (710 nodes, 3,044 sequences, and 71% motif coverage) and C5 (21 nodes, 39 sequences, and 54% motif coverage) clusters, the motif variant (FY)XGXXXHXXXF dominated the C4 cluster (56 nodes, 1,269 sequences, and 99% motif coverage), and the motif variant (FY)XGXXXHXXXH dominated the C3 cluster (75 nodes, 224 sequences, and 71% motif coverage). The latter observation may suggest that the FMO family signature motif should be expanded to include histidine in the last position, i.e., (FY)XGXXXHXXX(FYH).

To further focus the *in silico* screen for new bacterial Tmm candidates, we targeted the two FMO family sequence clusters that contained sequences annotated as Tmms. Tmm-annotated sequences were present in 32 of the 56 nodes of the strongly connected C4 cluster (alignment scores of 107 to 268, with a mean of 163) and in 2 of the 21 nodes of the smaller, more loosely connected C5 cluster (alignment scores of 50 to 212, with a mean of 78). Interestingly, 15 nodes from the C4 cluster contained only FMO-like family sequences, thus supporting the decision to not restrict the SSN analysis to the FMO family sequences. The abundance of Tmm-annotated sequences in the C4 cluster suggests that it may be an isofunctional cluster (23). We selected 38 sequences from the C4 cluster and 7 sequences from the C5 cluster for recombinant expression (Fig. 1; Table S1 in the supplemental material), forming a diverse set of 45 putative Tmm sequences from six distinct taxonomic groups: *Alphaproteobacteria* (9 sequences), *Betaproteobacteria* (2 sequences), *Gammaproteobacteria* (14 sequences), *Bacteroidetes* (8 sequences), *Actinobacteria* (9 sequences), and *Chloroflexi* (1 sequence), as well as two sequences of unclassified metagenomic origin. Three previously published Tmms for which structural information is available were included among the 45 selected sequences: mFMO from M. aminisulfidivorans strain SK1 (13, 14, 25), referred to as T10 in this study; RnTmm from R. nubinhibens ISM (15), referred to as T11; and NiFMO from Nitrincola lacisaponensis (21), referred to as T72.

**FIG 2:**
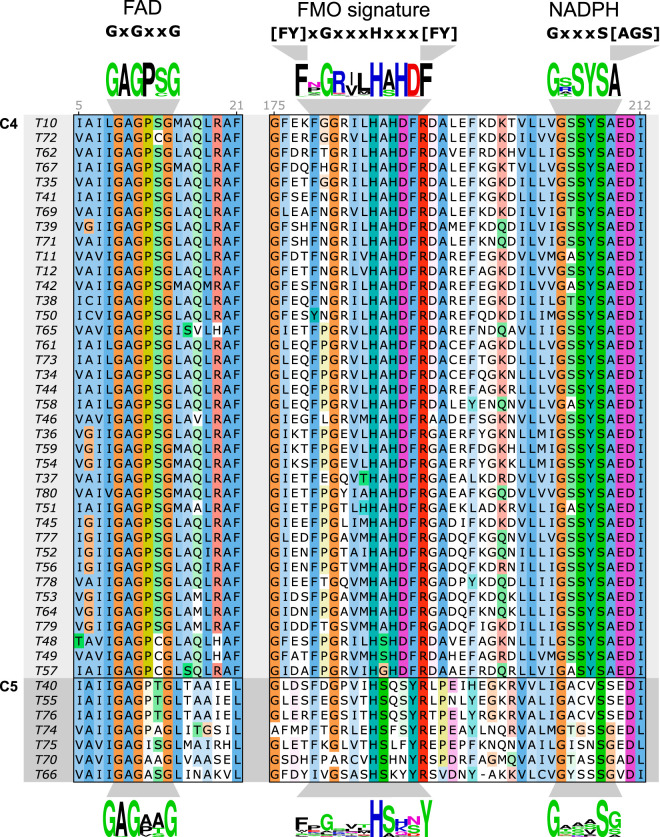
Conserved motifs in candidate Tmm sequences selected for recombinant expression. The figure shows two segments of a multiple-sequence alignment of the selected candidate Tmm sequences (each indicated by its arbitrary target ID number [T10 to T80] at the left) containing FAD-binding, NADPH-binding, and FMO signature sequence motifs. The sequences were grouped according to the SSN cluster to which they belong (C4 or C5) (Fig. 1), and the ClustalX color shading intensity was adjusted for each group by conservation, using a threshold of 30% sequence identity. The motifs shown at the top were informed by all bacterial FMO family (SSN clusters C2 to -5) (Fig. 1) sequences (x indicates that any residue is allowed, and any of the residues listed within brackets are allowed at that position). Sequence logos based on all C4 and C5 sequences are shown above and below the alignment, respectively. Sequence numbers for the T10 sequence (mFMO; UniProt accession number Q83XK4) are indicated in gray numbers above the alignment for reference.

In addition to the FMO signature motifs, the FMOs are characterized by their two dinucleotide binding motifs, involving FAD and NADPH binding. The FAD-binding motif, GXGXXG (26), is shared between FMOs and BVMOs, whereas the NADPH-binding motif, GXXXS[AGS], which was derived from the C2 to -5 sequences, is distinct to the FMOs. The FMO groups, as defined by the SSN clusters C2 to -5, show variations in their motif sequences, which is illustrated by motif sequence logos representing all Tmm candidate cluster C4 and C5 sequences (Fig. 2). The FAD-binding motif is well conserved in both clusters, the FMO signature motif is less conserved in C5 than in C4, and the NADPH-binding motif of C4, GXSYS[AGS], is highly conserved and is present in 99% of the C4 sequences. This motif includes a conserved tyrosine, which has been shown to act as a lid shielding the FAD intermediate from the solvent (13, 25) and may be a defining motif for bacterial Tmms. Interestingly, 98% of the C4 sequences match both the FMO signature variant motif, [FY]XGXXXHXXXF, and the NADPH-binding GXSYS[AGS] motif. A multiple-sequence alignment (MSA) of the selected Tmm candidate sequences demonstrated that the C4 sequences contained all three motifs, in contrast to the C5 sequences, where all sequences contained the dinucleotide binding motifs but only five of seven sequences contained the complete FMO signature motif (Fig. 2).

### Candidate expression, purification, and activity screening.

**FIG 3:**
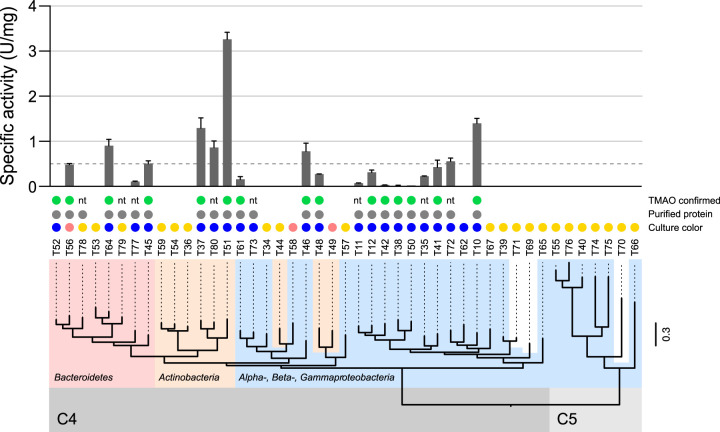
Candidate Tmm protein expression, purification, and activity data organized by evolutionary relationship. The phylogenetic tree (bottom) shows the evolutionary relationship between all 45 candidate Tmm sequences (indicated by arbitrary target ID numbers T10 to T80), and the two major clades, corresponding to SSN clusters C4 and C5, are indicated by gray shading. Major taxonomic phyla/classes are indicated by red (*Bacteroidetes*), yellow (*Actinobacteria*), and blue (*Alpha*-, *Beta*-, and *Gammaproteobacteria*) shading. T71 belongs to the *Chloroflexi*, and T69 and T70 are of metagenomic origin (unshaded). The branch lengths reflect expected substitutions per site (scale bar to the right). Bacterial culture colors after induction and expression of Tmm candidate proteins are indicated by blue, pink, and yellow dots (middle). Enzymes that were successfully purified by affinity chromatography are indicated by gray dots, and successful enzymatic TMAO product formation, as confirmed by CE-ESI-TOF-MS, is indicated by green dots (nt, not tested). *n* = 2 technical replicates. The bar graph (top) shows the specific activity (U/mg) against TMA for all purified enzymes (≥2 biological replicates each with ≥3 technical replicates), where 1 unit (U) of enzyme activity was defined as the amount of enzyme required to transform 1 μmol TMA in 1 min under the assay conditions using the reported NADPH extinction coefficient (ε_340_ = 6.22 mM*^−^*^1 ^cm*^−^*^1^). The dashed line indicates a specific activity of 0.5 U/mg. Error bars show standard deviations.

Each of the 45 candidate Tmms was expressed recombinantly in E. coli, fused to a C-terminal hexahistidine (6×His) for downstream purification. During expression in growth medium supplemented with the indole precursor l-tryptophan, we observed blue-colored medium for 21 candidate enzymes and pink coloration of the cell pellet for 3 others (Fig. 3). Tmms are known to catalyze the conversion of indole to the blue-colored pigment indigo (18, 21, 27), and some Tmms, such as T10 (22), are able to produce the pink-colored pigment indirubin (28), suggesting that the observed colors were the result of the enzymatic activity of the expressed Tmms.

SDS-PAGE analysis of the expressed Tmm candidates revealed that 31 of 45 appeared to be expressed in a soluble form (Table S1). These were subjected to small-scale Ni-nitrilotriacetic acid (NTA) spin column purification, and 23 were successfully purified at pH 7.5 (Fig. 3). The purified enzymes had a distinct yellow color, indicating that FAD was bound. The purified candidate enzymes were assessed for activity against TMA using the NADPH assay, which monitors decreasing absorbance at 340 nm resulting from the consumption of the cofactor NADPH (Fig. 3) (13, 25). Of the 23 candidate enzymes tested, 20 were shown to have specific activity against TMA, which was measured to be in the range of 0.01 to 3.25 U/mg (Fig. 3) (1 unit [U] of enzyme activity was defined as the amount of enzyme required to transform 1 μmol TMA in 1 min under the assay conditions using the reported NADPH extinction coefficient [ε_340_ = 6.22 mM^−1^ cm^−1^]). Eight enzymes (T10, T37, T45, T46, T51, T64, T72, and T80) demonstrated an activity of ≥0.5 U/mg under the experimental conditions (Fig. 3, dotted line), among which T51 showed the highest specific activity against TMA (3.25 U/mg). Interestingly, these top Tmm candidates were found in bacteria from four different taxonomic groups: *Actinobacteria* (T37, T51, and T80), *Bacteroidetes* (T45 and T64), *Gammaproteobacteria* (T10 and T72), and *Alphaproteobacteria* (T46).

To confirm that we observed *de facto* TMA oxidation, we measured TMAO product formation using capillary electrophoresis electrospray ionization time-of-flight mass spectrometry (CE-ESI-TOF-MS). Fifteen Tmm candidates were selected for CE-ESI-TOF-MS analysis, and all were shown to catalyze the production of TMAO (Fig. 3). These TMAO assay results corroborated the NADPH assay results (Fig. 3) and confirmed that the four candidates (T38, T42, T50, and T52) with the lowest specific activities in the NADPH assay (<0.03 U/mg) indeed were active. Moreover, the TMAO assay results verified that the NADPH assay reliably describes Tmm enzyme activities, and hence, the more convenient NADPH assay was used to conduct further biochemical characterizations of selected Tmms.

### Biochemical characterization of T10, T37, and T51.

The three Tmms with the highest specific activity against TMA in the NADPH assay were selected for further in-depth analysis to investigate their potential in industrial applications. These were the mFMO (T10) from M. aminisulfidivorans strain SK1 (13, 14, 25), T37 from Nocardiopsis alba, and T51 from Micrococcus terreus (Fig. 3; Table S1). Enzymatic processing of salmon by-products typically requires enzymes to perform at a pH of around 6 to 7 and temperatures of around 40 to 60°C, followed by enzyme inactivation by exposure to temperatures above 75°C (3, 29). Hence, to evaluate the industrial potential of the selected Tmms, we studied their pH and temperature profiles, both in terms of optimal enzyme activity and enzyme stability.

**FIG 4:**
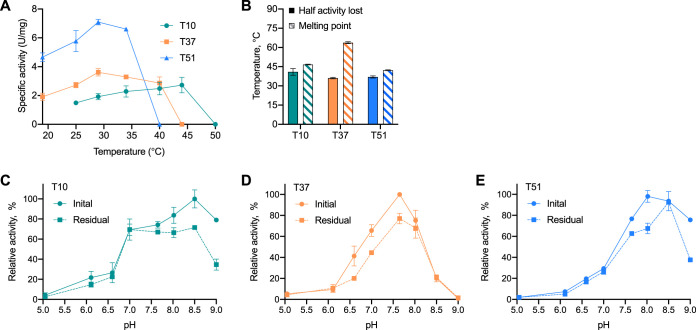
Biochemical characterization of Tmm enzymes T10, T37, and T51. (A) Specific activity (U/mg) against TMA at pH 7.5 measured across temperature (20 to 50°C) by NADPH consumption (*n* = 2 biological replicates, each with ≥2 technical replicates). One unit (U) of enzyme activity was defined as the amount of enzyme required to transform 1 μmol TMA in 1 min under the assay conditions using the reported NADPH extinction coefficient (ε_340_ = 6.22 mM*^−^*^1 ^cm*^−^*^1^). (B) Enzyme stability was studied by temperature-induced activity loss and protein unfolding. Activity loss was studied by performing 1-h incubations at temperatures from 30 to 54°C and measuring residual activity against TMA, and temperatures at which half the activity was lost were estimated by 4-parameter logistic regression (*n* = 3 biological replicates for T10, and *n* = 3 technical replicates for T37 and T51). Temperature-induced protein unfolding was monitored by circular dichroism, measured from 10°C to 95°C, and melting points were estimated by 4-parameter logistic regression (*n* = 3 technical replicates). (C to E) Initial and residual enzymatic activities of T10 (C), T37 (D), and T51 (E) after 2 h of incubation at the indicated pHs (Britton-Robinson buffers, pH 5.0 to 9.0) at 20°C. Activity is shown relative to maximal initial activity (100%) for each enzyme. For T10 and T37, there were 3 biological replicates, and for T51, there were 2 biological replicates with 3 technical replicates for initial activity; for all enzymes, *n* = 3 technical replicates for residual activity. Error bars show standard deviations.

To identify the optimal temperatures for enzymatic activity at pH 7.5, we measured the specific activities of the three Tmms between 20 and 50°C (Fig. 4A). The results show that T37 and T51 had comparable temperature activity profiles, which were distinct from that of T10. The recorded temperature optimum of T10 was 44°C, whereas the optimal temperature for both T37 and T51 was 29°C. No activity was observed at 40°C for T51, 44°C for T37, and 50°C for T10. To investigate to what extent incubation at different temperatures affected the enzyme activity, the Tmms were incubated for 1 h between 30 and 54°C and assessed for residual specific activity. As a measure of temperature stability, we performed a regression analysis of the resulting data to determine the temperature at which half the initial activity was lost (Fig. 4B, filled bars). Enzymatic activity declined at temperatures close to the optimal temperature for activity (Fig. 4A), with loss of half of the initial activity recorded at 41.2°C for T10, 36.1°C for T37, and 36.8°C for T51 (Fig. 4B, filled bars). To further investigate temperature stability, we performed temperature-induced protein unfolding experiments on each enzyme using circular dichroism (CD) and recorded the melting temperatures (*T_m_*) (Fig. 4B, striped bars). For T10 and T51, the *T_m_*s were found to be 46.7°C and 42.2°C, respectively, which is in agreement with the temperature-induced activity loss results (Fig. 4B, filled bars). In contrast, the *T_m_* for T37 was 63.7°C, much higher than the temperature at which half the activity was lost (36.0°C).

To identify the optimal pH ranges for the Tmms, defined as the range at which the activity was ≥50% of the maximum activity, we measured the activity between pH 5.0 and 9.0 at 20°C (Fig. 4C to E, solid lines). The activity was measured immediately after adding enzyme. The results show that T10 (Fig. 4C) has a broader pH profile (pH 7.0 to 9.0) than do T37 (pH 7 to 8) (Fig. 4D) and T51 (pH 7.5 to 9.0) (Fig. 4E). To investigate to what extent incubation at different pHs affected the enzyme activity over time, the enzymes were incubated from pH 5.0 to 9.0 for 2 h prior to measuring their residual activity. Of the three enzymes tested, T10 and T51 appeared relatively stable at industrial-process-relevant pH conditions of pH 6.0 to 7.0 (Fig. 4C and E). T37, on the other hand, appeared to be less stable under these pH conditions, with an observed activity drop of more than 20% compared to the initial activity (Fig. 4D). Although the pH optimum of activity is often correlated with the pH optimum of stability (30), the Tmm enzymes displayed more than 20% drops in activity around their optimal pHs (Fig. 4C to E).

### T10, T37, and T51 convert TMA to TMAO in a salmon protein hydrolysate.

**FIG 5:**
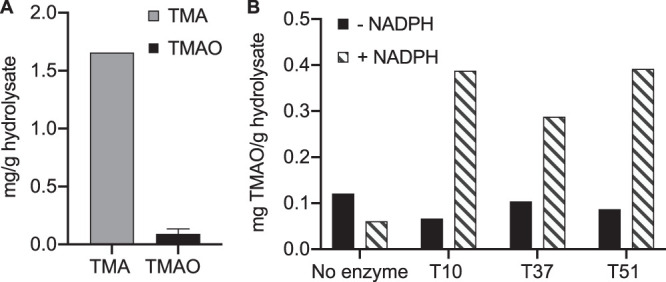
Tmm enzymes convert TMA to TMAO in salmon protein hydrolysate. (A) Levels of TMA and TMAO in salmon protein hydrolysate (wt/wt) in the absence of supplemental NADPH (for TMA, *n* = 1; for TMAO, *n* = 2). (B) Analysis of TMAO levels after Tmm-driven oxidation of TMA in salmon protein hydrolysate using CE-ESI-TOF-MS, in the presence (stripes) and absence (black) of 0.50 mM NADPH, after 1 h of incubation at 30°C with enzymes (*n* = 1). Salmon protein hydrolysate in the absence of Tmms served as a control (no enzyme) (*n* = 1).

As a proof of concept, we assessed the ability of T10, T37, and T51 to convert TMA to TMAO in a salmon protein hydrolysate. The initial levels of TMA and TMAO in the salmon protein hydrolysate were quantified by CE-ESI-TOF-MS as 1.6 mg/g hydrolysate and 0.12 mg/g hydrolysate, respectively (Fig. 5A). To test whether the Tmms could convert TMA to TMAO in the hydrolysate, enzymes were added and incubated in the presence or absence of 0.2 mM NADPH for 1 h at 30°C before determining TMAO concentrations using CE-ESI-TOF-MS. The results demonstrate that the enzymes were indeed capable of oxidizing TMA, given that a sufficient amount of NADPH was present, yielding increased amounts of TMAO in the salmon protein hydrolysate (Fig. 5B).

## DISCUSSION

In the present study, we aimed to identify Tmm enzymes capable of converting TMA to TMAO with high specific activity. Tmms may prove to be useful biocatalysts for transforming salmon protein hydrolysates into more desirable ingredients for human consumption, by oxidizing the malodorous TMA to the odor-neutral TMAO. The TMA levels in raw fish filets from Atlantic salmon stored at 4°C for zero to 2 days are reported to contain approximately 0.002 to 0.01 mg/g tissue (wet weight), which increases to 0.19 to 0.33 mg/g tissue after a week of storage at the same temperature (31, 32). TMA levels vary in the biomass depending on fish tissue, species, temperature, and processing (4, 31, 32). The protein hydrolysate made from Atlantic salmon applied in the present study had TMA levels of 1.6 mg/g hydrolysate (about 64.6% dry weight) (Fig. 5A), more than 160-fold the amount found in short-term cold-stored fish filet. These data suggest that all or a major fraction of the water-soluble TMA is retained in the soluble fraction during enzymatic hydrolysis and gives rise to increased TMA levels in the protein hydrolysates, which further emphasizes the challenge of achieving a desirable organoleptic quality. Accepted threshold levels of TMA in Atlantic salmon are reported to be 0.295 mg/g tissue (wet weight) (31), highlighting the need for novel strategies targeting this particular volatile odor molecule.

Previous attempts to solve problems with undesired odors of marine hydrolysates include odor masking. Salt-water clam hydrolysates have been treated with tea polyphenol (33), which masked the odor by affecting TMA, and Atlantic salmon hydrolysates have been treated by adding sugars that proved effective, despite not affecting TMA (34). The sugar-treated salmon hydrolysates, however, had a distinct grilled odor, which may not be desirable. In contrast to odor masking, we have shown that the Tmms, such as T10, T37, and T51, can be used to remove TMA from such hydrolysates (Fig. 5A), possibly avoiding unwanted side effects.

In the proof-of-concept experiment performed on a salmon hydrolysate, enzymatic activity against TMA was only observed after the cofactor NADPH was added to the hydrolysates. Other TMA-rich fish products may have sufficient intrinsic levels of NADPH to drive Tmm enzymatic activity, but where that is not the case, cofactor dependency must be addressed. NADPH is an expensive chemical, and hence, adding NADPH to an industrial process is highly unlikely to be cost effective. One solution is to engineer the enzymes to accept alternative cofactors (35), which may be present in the TMA-rich biomaterial of interest or are less expensive to add. Another solution is to regenerate the cofactor (36, 37). Flavin binding monooxygenases, such as class I type I BVMOs, have been fused to a glucose dehydrogenase to obtain a continuous oxidation reaction driven by the dehydrogenase-catalyzed regeneration of NADPH from NADP^+^ (38).

The three Tmms that were subjected to biochemical characterization have different temperature and pH profiles and can thus serve as unique starting points for enzyme engineering to tailor the enzymes for industrial applications. Among our panel of enzymes, the mFMO (T10) from M. aminisulfidivorans may be the best candidate for the process used to generate the salmon hydrolysate studied here, which operates at 40 to 60°C and pH 6.0 to 7.0 (3, 29). Still, engineering is likely to be required to increase the enzyme stability and temperature optimum. In contrast, for processes that operate at lower temperatures, T37 from N. alba and T51 from M. terreus are likely to be more suited and better starting points for engineering, if required. Finally, the Tmms identified here demonstrate that there are Tmms in several taxonomic groups, including *Actinobacteria*, *Bacteroidetes*, *Gammaproteobacteria*, and *Alphaproteobacteria*, that could be further explored for their industrial applicability.

## MATERIALS AND METHODS

### SSN construction.

A sequence similarity network (SSN) was created for the InterPro flavin monooxygenase-like (FMO-like) family (InterPro accession number IPR020946) using the Enzyme Function Initiative Enzyme Similarity Tool (EST-EFI) (23). The SSN was constructed using UniRef90 and default settings, and the SSN was finalized using an alignment score threshold of 35 and only including sequences with a length of 100 to 800 amino acids. Due to the large size of the network, a representative network, where each node is a collection of proteins with 40% or higher identity, was downloaded from the server and processed further using Cytoscape 3.8.0 (39). Cytoscape was used to remove nodes containing only nonbacterial sequences and edges with alignment scores lower than 50 and to visualize the final SSN using the yFiles organic layout.

### MSA and phylogenetic tree construction.

A multiple-sequence alignment (MSA) of the Tmm candidate sequences was made using Clustal X (40) with default parameters. The alignment was visualized with JalView 2.11.1.0 (41), and sequence logos were made using WebLogo (42). The MSA was used to construct a phylogenetic tree with MrBayes version 3.2.6 (Ronquist et al., 2012), using the following settings: the priors for the amino acid model were set to mixed, and the number of generations used was 200,000. The resulting phylogenetic tree was visualized with FigTree version 1.1.3, and the sequences from the SSN C5 cluster were used as an outgroup to root the tree.

### Molecular cloning of candidate enzymes.

All candidate enzymes were ordered as DNA from Twist Bioscience (San Francisco, CA, USA) and subcloned and sequenced as previously described (43, 44). Briefly, codon-optimized genes flanked by SapI sites were subcloned by fragment exchange (FX) cloning into the pBXC3H vector containing a C-terminal 6×His tag. The ligation reaction product was transformed into E. coli MC1061 cells, and clones were selected on LB agar (1% [wt/vol] tryptone, 0.5% [wt/vol] yeast extract, 1% [wt/vol] NaCl, 1.5% [wt/vol] agar-agar) supplemented with kanamycin (50 μg/ml; Sigma-Aldrich). Plasmids were isolated using the NucleoSpin plasmid purification kit (Macherey-Nagel), and Sanger sequencing was used to confirm correct cloning.

### Expression and solubility screening.

All enzyme constructs were expressed in E. coli MC1061 cells in 24-well deep-well plates in LB medium (Sigma-Aldrich) supplemented with 0.1% (wt/vol) l-tryptophan (Sigma-Aldrich) and 100 μg/ml ampicillin at both 20°C and 30°C for 16 h after induction with l-arabinose (Sigma-Aldrich) to a final concentration of 1% (wt/vol). Proteins were purified using Ni-NTA spin columns (Qiagen), following the Qiagen protocol. Cells were collected by spinning at 4,000 × *g* for 30 min at 4°C and lysed in lysis buffer (50 mM Tris-HCl, pH 7.5, 500 mM NaCl, 1 mM phenylmethanesulfonyl fluoride [Sigma-Aldrich], 0.1% *n*-dodecyl β-d-maltoside [Sigma-Aldrich], 5 μg/ml DNase [Sigma-Aldrich], 0.2 mg/ml lysozyme [Sigma-Aldrich]). Columns were equilibrated with equilibration buffer (50 mM Tris-HCl, pH 7.5, 500 mM NaCl, 10 mM imidazole) before incubation with cell lysate and washed with wash buffer (50 mM Tris-HCl, pH 7.5, 500 mM NaCl, 30 mM imidazole). Proteins were eluted with elution buffer (50 mM Tris-HCl, pH 7.5, 500 mM NaCl, 500 mM imidazole). Total protein fractions of cell lysate, soluble fractions, and eluted proteins were analyzed by SDS-PAGE (Bio-Rad) (Fig. S1 in the supplemental material).

### Medium-scale protein expression and purification.

Soluble candidate enzymes were expressed in E. coli MC1061 cells in 200 ml LB medium supplemented with 0.1% (wt/vol) l-tryptophan at 20°C or 30°C for 16 h after induction with l-arabinose to a final concentration of 1% (wt/vol). All subsequent steps were carried out at 4°C. Cells were collected by centrifugation at 19,000 rpm for 20 min. The cell pellets were resuspended in lysis buffer and subjected to three freeze-thaw cycles, including 10 min on dry ice and ethyl alcohol (EtOH), followed by 15 min in a room temperature water bath. Following freeze-thawing, the cell lysate was sonicated briefly 3 times for 10 s at 60% amplitude to remove DNA. The cell lysate was finally spun at 17,000 rpm for 30 min, and the cleared lysate was passed over preequilibrated prepacked Ni-NTA columns (Thermo Fisher). The resin was washed with 10 column volumes of wash buffer before elution by flowing elution buffer over the Ni-NTA columns. The eluted protein was then subjected to buffer exchange using equilibrated (50 mM Tris-HCl, pH 7.5, 100 mM NaCl) PD10 columns (GE Healthcare). Protein concentrations were measured using the Bradford method. Enzyme purity was checked by SDS-PAGE (Fig. S1). The eluted proteins were stored with 10% glycerol at −20°C until further use.

### NADPH assay.

Activity assessments were conducted by detecting the conversion of NADPH to NAD^+^ at 340 nm in 96-well plates. All assays were carried out in reaction buffer (50 mM Tris-HCl, pH 7.5, 100 mM NaCl) supplemented with 0.25 to 0.5 mM NADPH (Sigma-Aldrich) and 0.01 to 0.02 mg/ml enzyme. Reactions were initiated by the addition of 1 mM TMA (Sigma-Aldrich). We assumed the concentrations of TMA and NADPH to be saturating (>10× *K_m_*) for all enzymes to be tested based on previous publications on T10 (*K_m_*_, TMA_ = 7.3 μM and *K_m_*_, NADPH_ = 13.0 μM) (13) and T72 (*K_m_*_, TMA_ = 45.6 μM and *K_m_*_, NADPH_ = 8.2 μM) (21). The decrease in absorbance at 340 nm was measured continuously over 30 min by UV-visible (UV-Vis) spectrophotometry in a Synergy HT multi-mode microplate reader. The initial rates were determined from linear fits of the absorbance versus time and corrected by the results for the blank. All assays (performed in triplicates) were performed at 21°C unless stated otherwise. In all cases, 1 unit (U) of enzyme activity was defined as the amount of enzyme required to transform 1 μmol substrate in 1 min under the assay conditions using the reported extinction coefficient (ε_340_ = 6.22 mM^−1 ^cm^−1^ for NADPH).

### pH optimum and stability.

pH optimum and stability were measured by the NADPH assays as described above, using Britton-Robinson buffer (pH 5.0 to 9.0) (45) instead of reaction buffer. For stability assays, enzymes were diluted to 0.5 mg/ml in the various buffers and incubated at 20°C for 2 h before measuring residual enzymatic activity as described above. Relative pH stability was plotted (GraphPad Prism 8) by setting the optimum of each enzyme as 100%.

### Temperature optimum.

The temperature optimum was measured using indirect enzymatic assays as described above but using 2-ml cuvettes and an Agilent 8453 UV-Vis spectrophotometer connected to a circulating water bath. Specific enzyme activities were determined at temperatures from 20°C to 60°C in intervals of 5°C. Reaction mixtures contained temperature-equilibrated reaction buffer (50 mM Tris-HCl, pH 7.5, 100 mM NaCl), 0.25 mM NADPH, and 1 mM TMA. Reactions were started by adding 0.01 to 0.02 mg/ml purified enzyme and continuously monitored at 340 nm over 5 min.

### Temperature stability.

Freshly purified enzymes were diluted to 1.0 mg/ml in reaction buffer (50 mM Tris-HCl, pH 7.5, 100 mM NaCl) and the initial enzyme activity was measured as described above. Enzymes were next incubated for 1 h at temperatures from 30°C to 54°C using a PCR thermocycler machine (Bio-Rad). After incubation, samples were cooled for 10 min at 4°C, followed by 10 min at room temperature before spinning for 2 min using a small tabletop centrifuge. Residual enzyme activity was measured as described above and was plotted by setting the initial activity as 100%. The temperature where half of the activity was lost was found by 4-parameter logistic regression using GraphPad Prism8.

### CD spectroscopy.

Melting temperatures were recorded for enzymes diluted to 0.6 mg/ml. Circular dichroism (CD) spectra were acquired between 190 and 270 nm with a Jasco J-720 spectropolarimeter equipped with a Peltier temperature controller, employing a 0.1-mm cell. Spectra were analyzed, and denaturation temperatures (*T_d_*) were determined at 220 nm, between 10 and 95°C, at a rate of 30°C per h in reaction buffer (50 mM Tris-HCl, pH 7.5, 100 mM NaCl). *T_d_* (and standard deviation of the linear fit) was calculated by fitting the ellipticity (millidegrees [mdeg]) at 220 nm at each of the different temperatures using 4-parameter logistic regression (*n* = 3 technical replicates).

### Capillary electrophoresis electrospray ionization time-of-flight mass spectroscopy (CE-ESI-TOF-MS) sample preparation.

Enzymatic reactions were set up in duplicates in 50-kDa filters in volumes of 0.3 ml containing assay buffer, 0.25 mM NADPH, and 5 to 10 ng/ml enzyme at 30°C and 500 rpm. Reactions were initiated by the addition of 1.0 mM TMA. The filters were spun at 14,000 × *g* for 2 min at room temperature to stop the reaction. The flowthrough was collected, diluted 1:1 with a mixture of H_2_O-acetonitrile (95:5), 0.2 M formic acid (CH_2_O_2_), and 0.4 mM methionine sulfone (MeSO_4_), and vortexed for 5 min. Samples were stored at −80°C until analysis. To quantify the TMA and TMAO, two series of calibration samples were prepared by spiking different quantities of TMA (concentration range in the final sample, 10, 25, 50, and 100 mg/liter) and TMAO (0, 5, 10, 25, and 50 mg/liter).

Salmon protein hydrolysate (64.4% dry weight; provided by Biomega Group) was produced from fresh salmon by-products processed with proteases. The hydrolysate was diluted 1:5 (wt/vol) in ultrapure water due to its viscous nature in order to be able to analyze the solution using CE-ESI-TOF-MS. Diluted hydrolysate was sonicated in a water bath at 50 Hz for 5 min and vortexed for 5 min before centrifugation at 16,000 × *g* for 10 min and collection of the supernatant (SPN). The pH of the SPN was 6.10. Samples were prepared with SPN in the presence or absence of 0.50 mM supplemental NADPH in addition to 10 ng/ml enzyme and incubated for 1 h at 30°C. After incubation, samples were filtered using 50-kDa-cutoff filters (Amicon) to remove enzymes. Flowthrough was collected, diluted 1:1 with a mixture of H_2_O-AcN (95:5), 0.2 M formic acid (CH_2_O_2_), and 0.4 mM methionine sulfone (MeSO_4_), and stored at −80°C until analysis. Dilute salmon protein hydrolysate without enzymes was prepared in a similar fashion and used as a control. To quantify the TMA and TMAO in the salmon protein hydrolysate samples, another two series of calibration samples were prepared by spiking different quantities of TMA (50, 100, and 200 mg/liter) and TMAO (5, 10, 25, and 50 mg/liter).

### CE-ESI-TOF-MS target analysis.

The data were obtained by CE (7100 Agilent) coupled to TOF (6224 Agilent). The separations occurred in a fused-silica capillary (total length, 100 cm, and inner diameter, 50 μm; Agilent). All separations were performed in normal polarity with a background electrolyte containing 1.0 M formic acid (FA) in 10% methanol (MeOH) (vol/vol) at 20°C. New capillaries were preconditioned with a flush of 1.0 M NaOH for 30 min, followed by MilliQ water for 30 min and the background electrolyte for 30 min. Before each analysis, the capillary was conditioned with a 5-min flush of the background electrolyte. The sheath liquid (0.6 ml/min) was MeOH-H_2_O (1:1) containing 1.0 mM FA with one reference mass of 121.0509 (purine, detected *m/z* [C_5_H_4_N_4_ + H]^+^), which allowed for correction and higher mass accuracy in the MS. The samples were hydrodynamically injected at 5,000 Pa for 17 s. Stacking was performed by applying the background electrolyte at 10,000 Pa for 10 s. The separation voltage was 30 kV, the internal pressure was 2,500 Pa, and the analyses were performed during 25 min. The MS parameters were as follows: fragmentor voltage, 125 V; skimmer voltage, 65 V; octopole voltage, 750 V; drying gas temperature, 200°C; flow rate, 10 liters/min; and capillary voltage, 3,500 V. The data were acquired in positive mode with a full scan from *m/z* 50 to 500 at a rate of 1.00 scan per second. Samples were analyzed in randomized runs. The analytical run was set up starting with the analysis of 5 injections of a pool of samples to equilibrate the system, followed by injections of the calibration samples and then the samples in a randomized order. After the final samples were injected, the series of calibration samples were injected again. The enzymatic reactions and salmon protein hydrolysate samples were analyzed in two independent analytical runs.

The corresponding peak areas were integrated using MassHunter Quantitative Analysis (B.09.00; Agilent). The final concentration per sample was calculated based on the peak area for the corresponding standard in a calibration curve. The linearity of the relative response versus concentration was previously assessed under the same analytical conditions (for enzymatic reactions, *r*^2^ = 0.9991 for TMA and *r*^2^ = 0.9944 for TMAO, and for salmon protein hydrolysate samples, *r*^2^ = 0.9973 for TMA and *r*^2^ = 0.9975 for TMAO).

## Supplementary Material

Supplemental file 1
