# Supplementary material for: Use of Flavin-Containing Monooxygenases for Conversion of Trimethylamine in Salmon Protein Hydrolysates
Source: Appl Environ Microbiol. 2020 Nov 24;86(24):e02105-20. doi: 10.1128/AEM.02105-20 (PMC7688232; doi:10.1128/AEM.02105-20)
Supplement: Supplemental file 1 [file AEM.02105-20-s0001.pdf]

Table S1. Overview of Tmm sequences investigated and summary of results from expression, purification and functional analyses.

Candidate[a]	UniProt acc.no	Taxonomic class	Organism	Indigo	Pink	Expressing	Soluble	Purified	TMA active[b]	Specific activity[c]	Cluster
T10	Q83XK4	Gammaproteobacteria	Methylophaga aminisulfidivorans	+	-	+	+	+	+	1.40	C4
T11	A3SLM3	Alphaproteobacteria	Roseovarius nubinhibens (strain ATCC BAA-591 / DSM 15170 / ISM)	+	-	+	+	+	+	0.07	C4
T12	Q5LT63	Alphaproteobacteria	Ruegeria pomeroyi (strain ATCC 700808 / DSM 15171 / DSS-3) (Silicibacter pomeroyi)	+	-	+	+	+	+	0.31	C4
T34	A0A024E3V8	Gammaproteobacteria	Pseudomonas mandelii JR-1	-	-	-	-	-	NT		C4
T35	A0A2N7TXS4	Gammaproteobacteria	Halomonas endophytica	+	-	+	+	+	+	0.23	C4
T36	H0QS70	Actinobacteria	Arthrobacter globiformis NBRC 12137	-	-	-	-	-	NT		C4
T37	J7LJI0	Actinobacteria	Nocardiopsis alba (strain ATCC BAA-2165 / BE74)	+	-	+	+	+	+	1.29	C4
T38	A0A0L1JP69	Alphaproteobacteria	Pseudaestuariivita atlantica	+	-	+	+	+	+	0.02	C4
T39	A0A0M4M1X0	Gammaproteobacteria	Candidatus Thioglobus singularis PS1	-	-	-	-	-	NT		C4
T40	A0A1H0Z7A2	Alphaproteobacteria	Pseudovibrio sp. Tun.PSC04-5.I4	-	-	+	+	-	NT		C5
T41	A0A1C9BF40	Gammaproteobacteria	Cobetia marina (Deleya marina)	+	-	+	+	+	+	0.43	C4
T42	A0A1W2EFV5	Alphaproteobacteria	Pseudooceanicola flagellatus	+	-	+	+	+	+	0.03	C4
T44	A0A1G7DRH8	Actinobacteria	Rhodococcus tukisamuensis	-	-	-	-	-	NT		C4
T45	A0A1Q5PDZ1	Bacteroidetes/ Chlorobi group	Pontibacter sp. S10-8	+	-	+	+	+	+	0.5	C4

T46	A0A1S2DRZ0	Alphaproteobacteria	Agrobacterium vitis (Rhizobium vitis)	+	-	+	+	+	+	0.78	C4
T48	A0A172UQK4	Actinobacteria	Mycobacterium sp. YCRL4	+	-	+	+	+	+	0.27	C4
T49	A0A1S8C9I2	Actinobacteria	Modestobacter sp. VKM Ac-2676	-	+	+	-	-	NT		C4
T50	A0A1X6ZIZ2	Alphaproteobacteria	Roseovarius halotolerans	+	-	+	+	+	-	0.02	C4
T51	A0A1I7MIR7	Actinobacteria	Micrococcus terreus	+	-	+	+	+	+	3.26	C4
T52	A0A1M6DLQ6	Bacteroidetes/ Chlorobi group	Aequorivita viscosa	+	-	+	+	+	-	0.001	C4
T53	A0A1X7IIE6	Bacteroidetes/ Chlorobi group	Sphingobacterium psychroaquaticum	-	-	+	+	-	NT		C4
T54	A0A1H8M3A0	Actinobacteria	Cryobacterium luteum	-	-	-	-	-	NT		C4
T55	G8PW09	Alphaproteobacteria	Pseudovibrio sp. (strain FO-BEG1)	-	-	+	+	-	NT		C5
T56	A0A0X7BCI4	Bacteroidetes/ Chlorobi group	Flavobacterium sp. TAB 87	-	+	+	+	+	+	0.48	C4
T57	A0A2E7T993	Gammaproteobacteria	Acidiferrobacteraceae bacterium	-	-	+	+	-	NT		C4
T58	A0A1Y0D4G1	Gammaproteobacteria	Oceanisphaera profunda	-	+	+	+	-	NT		C4
T59	A0A0M4MBH5	Actinobacteria	Arthrobacter sp. ERGS1:01	-	-	+	-	-	NT		C4
T61	A0A1D2RYI9	Betaproteobacteria	Acidovorax sp. SCN 6822	+	-	+	+	+	+	0.16	C4
T62	A0A157SFL4	Betaproteobacteria	Bordetella trematum	+	-	+	+	-	NT		C4
T64	F0S6P1	Bacteroidetes/ Chlorobi group	Pseudopedobacter saltans (strain ATCC 51119 / DSM 12145 / JCM 21818 / LMG 10337 / NBRC 100064 / NCIMB 13643) (Pedobacter saltans)	+	-	+	+	+	+	0.90	C4
T65	A0A081K627	Gammaproteobacteria	Endozoicomonas elysicola	-	-	-	-	-	NT		C4

T66	F8G8N2	Gammaproteobacteria	Francisella salina	-	-	-	-	-	NT		C5
T67	A0A1E8DZ78	Gammaproteobacteria	Acinetobacter towneri	-	+	-	-	-	NT		C4
T69	A0A160TRA3	hydrothermal vent metagenome	hydrothermal vent metagenome	-	-	-	-	-	NT		C4
T70	A0A160TR55	hydrothermal vent metagenome	hydrothermal vent metagenome	-	-	+	-	-	NT		C5
T71	A0A2E7WDS6	Chloroflexi	Chloroflexi bacterium	-	-	+	-	-	NT		C4
T72	A0A063Y6V3	Gammaproteobacteria	Nitrincola lacisaponensis	+	-	+	+	+	+	0.56	C4
T73	A0A0A0ESM2	Gammaproteobacteria	Lysobacter concretionis Ko07 = DSM 16239	+	-	+	+	+	-		C4
T74	A0A142B6Q7	Gammaproteobacteria	Endozoicomonas montiporae CL-33	-	-	+	-	-	NT		C5
T75	A0A1X7AJG6	Gammaproteobacteria	Parendozoicomonas haliclonae	-	-	+	+	-	NT		C5
T76	A0A165VSW0	Alphaproteobacteria	Pseudovibrio axinellae	-	-	+	+	-	NT		C5
T77	A0A1M6IW29	Bacteroidetes/ Chlorobi group	Arenibacter nanhaiticus	+	-	+	+	+	+	0.11	C4
T78	A0A2K8XUM8	Bacteroidetes/ Chlorobi group	Polaribacter sp. ALD11	-	-	+	+	+	-	0.004	C4
T79	F0P312	Bacteroidetes/ Chlorobi group	Weeksella virosa (strain ATCC 43766 / DSM 16922 / JCM 21250 / NBRC 16016 / NCTC 11634 / CL345/78)	-	-	+	+	+	-		C4
T80	A0A2W2K8B4	Actinobacteria	Streptomyces sp. NTH33	+	-	+	+	+	+	0.86	C4

a, Structural information is available for T10 (1), T11 (2), and T72 (3).

b, NT = not tested

c, SA, Specific activity in U/mg at pH 7.5, RT

Fig. S1

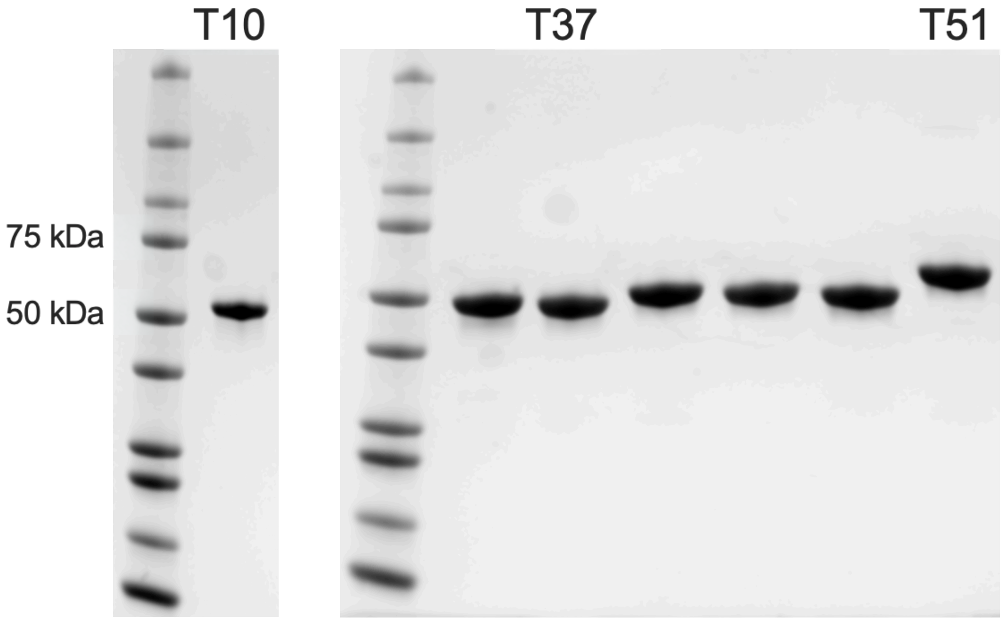

Fig. S1. SDS-PAGE (4-12%) of Tmm candidates T10, T37 and T51 after Ni-NTA purification and buffer exchange using PD10 columns. 2 µg of each sample was loaded on the gel.

References

1. Alfieri A, Malito E, Orru R, Fraaije MW, Mattevi A. 2008. Revealing the moonlighting role of NADP in the structure of a flavincontaining monooxygenase. Proceedings of the National Academy of Sciences 105:6572-6577.
2. Li C-Y, Chen X-L, Zhang D, Wang P, Sheng Q, Peng M, Xie B-B, Qin Q-L, Li P-Y, Zhang X-Y, Su H-N, Song X-Y, Shi M, Zhou B-C, Xun L-Y, Chen Y, Zhang Y-Z. 2017. Structural mechanism for bacterial oxidation of oceanic trimethylamine into trimethylamineNoxide. Molecular Microbiology 103:992-1003.
3. Loncar N, Fiorentini F, Bailleul G, Savino S, Romero E, Mattevi A, Fraaije MW. 2019. Characterization of a thermostable flavincontaining monooxygenase from Nitrincola lacisaponensis (NiFMO). Appl Microbiol Biotechnol 103:1755-1764.
